# Vertical Transmission of SARS-CoV-2: A Systematic Review

**DOI:** 10.1055/s-0040-1722256

**Published:** 2021-04-15

**Authors:** Ionara Diniz Evangelista Santos Barcelos, Ivan Andrade de Araújo Penna, Adriana de Góes Soligo, Zelma Bernardes Costa, Wellington Paula Martins

**Affiliations:** 1 Department of Obstetrics and Gynecology, Universidade Estadual do Oeste do Paraná, Cascavel, Paraná, PR, Brazil; 2 Department of Maternal-Infant Care, Universidade Federal Fluminense, Niterói, Rio de Janeiro, RJ, Brazil; 3 Private Clinic of Human Reproduction Dra. Adriana de Góes, São Paulo, SP, Brazil; 4 Department of Obstetrics and Gynecology, Universidade Federal de Goiás, Goiânia, GO, Brazil; 5 SEMEAR Fertilidade, Ribeirão Preto, São Paulo, SP, Brazil

**Keywords:** vertical transmission, SARS-CoV-2, COVID-19, perinatal outcomes, maternal morbidity, transmissão vertical, SARS-CoV-2, COVID-19, resultados perinatais, morbidade materna

## Abstract

**Objective** The evaluation of the available evidence on vertical transmission by severe acute respiratory syndrome coronavirus 2 (SARS-CoV)-2.

**Data Sources** An electronic search was performed on June 13, 2020 on the Embase, PubMed and Scopus databases using the following search terms: ( *Coronavirus* OR *COVID-19* OR *COVID19* OR *SARS-CoV-2* OR *SARS-CoV2* OR *SARSCoV2* ) AND ( *vertical* OR *pregnancy* OR *fetal* ).

**Selection of Studies** The electronic search resulted in a total of 2,073 records. Titles and abstracts were reviewed by two authors (WPM, IDESB), who checked for duplicates using the pre-established criteria for screening (studies published in English without limitation regarding the date or the status of the publication).

**Data Collection** Data extraction was performed in a standardized way, and the final eligibility was assessed by reading the full text of the articles. We retrieved data regarding the delivery of the potential cases of vertical transmission, as well as the main findings and conclusions of systematic reviews.

**Data Synthesis** The 2,073 records were reviewed; 1,000 duplicates and 896 clearly not eligible records were excluded. We evaluated the full text of 177 records, and identified only 9 suspected cases of possible vertical transmission. The only case with sufficient evidence of vertical transmission was reported in France.

**Conclusion** The risk of vertical transmission by SARS-CoV-2 is probably very low. Despite several thousands of affected pregnant women, we have identified only one case that has fulfilled sufficient criteria to be confirmed as a case of vertical transmission. Well-designed observational studies evaluating large samples are still necessary to determine the risk of vertical transmission depending on the gestational age at infection.

## Resumo

**Objetivo** Avaliar a evidência disponível acerca da transmissão vertical do coronavírus da síndrome respiratória aguda grave 2 (severe acute respiratory syndrome coronavirus 2, SARS-CoV-2, em inglês).

**Fontes de Dados** Foi realizada uma busca eletrônica em 13 de junho de 2020 nas plataformas Embase, PubMed e “Scopus utilizando os seguintes termos de busca: ( *Coronavirus* OU *COVID-19* OU *COVID19* OU *SARS-CoV-2* OU *SARS-CoV2* OU *SARSCoV2* ) E ( *vertical* OU *pregnancy* OU *fetal* ).

**Seleção dos Estudos** A busca eletrônica resultou em um total de 2.073 registros. Títulos e resumos foram revisados por dois autores (WPM, IDESB), que verificaram a ocorrência de duplicidade e utilizaram critérios preestabelecidos para o rastreamento (estudos publicados em inglês sem limitações quanto à data ou à situação da publicação).

**Aquisição dos Dados** A extração de dados foi realizada de forma padrão, e a eligibilidade final foi definida poir meio da leitura do artigo completo. Foram coletados dados dos partos de casos com potencial transmissão vertical, bem como os principais achados e conclusões de revisões sistemáticas.

**Síntese dos Dados** Foram revisados os 2.073 registros; 1.000 duplicatas e 896 registros claramente não elegíveis foram excluídos. Avaliamos os artigos completos de 177 registros, e identificamos apenas 9 casos de potencial transmissão vertical. O único caso com evidência suficiente de transmissão vertical foi relatado na França.

**Conclusão** O risco de transmissão vertical pelo vírus SARS-CoV-2 é provavelmente muito baixo. Apesar de milhares de gestantes afetadas, identificamos apenas um caso que preencheu critérios suficientes para que fosse confirmado como um caso de transmissão vertical. Estudos observacionais bem desenhados que avaliem grandes amostras ainda são necessários para se determinar o risco de transmissão vertical, a depender da idade gestacional na infecção.

## Introduction

At the end of 2019, a new virus was discovered: SARS-CoV-2. It first emerged in China, in the city of Wuhan, and quickly spread throughout the world, causing the coronavirus-19 disease (COVID-19). 1 This virus transmits extraordinarily rapidly. Therefore, pregnant women have become a concern, given their susceptibility to respiratory infections, due to the physiological changes during pregnancy and the restriction of lung expansion. 2 3

The current coronavirus (SARS-CoV-2), shares many structural similarities with other coronaviruses, like SARS-CoV and Middle East respiratory syndrome coronavirus (MERS-CoV). However, SARS-CoV-2 is less virulent, and its performance, as well as that of SARS-CoV, is mediated by the angiotensin-converting enzyme 2 (ACE2) receptor, a component of the renin-angiotensin system present in the lungs, heart, kidneys, and placenta. 4 5

Affinity with the receptor determines the route of the viral infection, and its identification in the placenta alerts to the possibility of vertical transmission. 6 Although present, the link between SARS-CoV-2 and the ACE2 receptor in the placenta is poorly expressed, which can be a protective factor for vertical transmission. Several studies have been published in recent months describing viral behavior in pregnant women and newborns. However, the impact of COVID-19 during pregnancy and the neonatal period is not yet fully supported by scientific research. 1

A better understanding of the viral pathogenesis in the pregnancy cycle is necessary to enable the monitoring of this group who is considered susceptible to this infection. The present review aims to identify the available evidence regarding the risk of vertical transmission by SARS-CoV-2 to guide family, gestational, and perinatal planning.

## Methods

### Eligibility Criteria

Observational studies with suspected vertical transmission and systematic reviews assessing the risk of vertical transmission were considered eligible.

### Search and Selection of the Studies

We searched the PubMed, Scopus, and Embase databases using the following search terms: ( *Coronavirus* OR *COVID-19* OR *COVID19* OR *SARS-CoV-2* OR *SARS-CoV2* OR *SARSCoV2* ) AND ( *vertical* OR *pregnancy* OR *fetal* ). Titles and abstracts were reviewed by two authors (WPM, IDESB), who checked for duplicates using the pre-established criteria for screening. We limited the search to studies published in English, but there was no limitation regarding the date or the status of the publication. After screening, the full texts of the records considered potentially eligible were retrieved for the final evaluation of eligibility.

### Data Collection/Extraction

Data extraction was performed in a standardized way, and final eligibility was assessed by reading the full text of the articles. The studies were characterized according to their design. We retrieved data regarding the delivery of the potential cases of vertical transmission, as well as the main findings and conclusions of systematic reviews and the conclusions from other reviews. The extracted data are presented in tables.

## Results

**Fig. 1:**
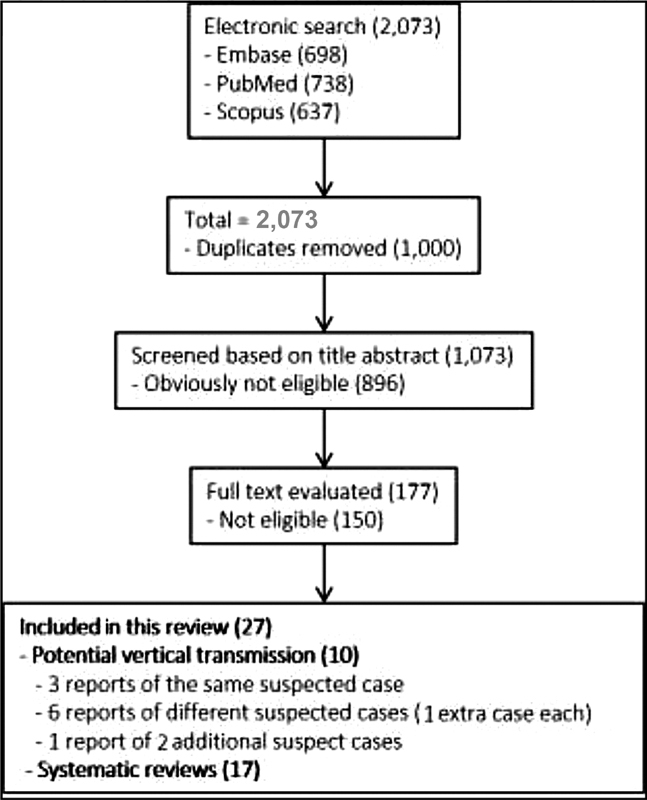
Flowchart of the selection of studies.

The last electronic search was performed on June 13, 2020, resulting in a total of 2,073 records from the 3 databasis consulted: Embase ( *n* = 698), PubMed ( *n* = 738), and Scopus ( *n* = 637). We excluded 1,000 duplicates, and 1,073 records were screened based on titles and abstracts, resulting in the exclusion of 896 records, as they were not related to the vertical transmission of COVID-19. We evaluated the full text of 177 records ( Fig. 1 ); out of those, we considered the following studies eligible:

Nine cases of potential vertical transmission were described in ten reports ( Table 1 ).

- Three reports of the same case of a potential vertical transmission at Tongji Hospital, Wuhan, China. 7 8 9
- One report of a potential vertical transmission at Renmin Hospital, Wuhan, China. 10
- One report of a potential vertical transmission at Imam Khomeini Hospital, Sari, Iran. 11
- One report of two suspect cases at hospital maternity units of the COVID-network in Lombardy and units of Padua and Modena, northern Italy. 12
- One report of a potential vertical transmission at the British American Hospital, Lima, Peru. 13
- One report of a potential vertical transmission at Paris Saclay University Hospitals, France. 14
- One report of a potential vertical transmission at Saint Barnabas Medical Center, United States. 15
- One report of potential vertical transmission at Henan Provincial People's Hospital, China. 16

**Table 1:** Reports of potential vertical transmission

Case	Country; city; hospital	Age (years)	Gestational age	Birth weight	Apgar score	Delivery	Main findings	Interval between onset of maternal disease and diagnosis in the neonate	Reasons why vertical transmission is not justified (or justified)
#1 Wang et al., 7 Hu et al., 8 Yu et al. 9	China; Wuhan; Tongji Hospital	34	39 weeks 6 days	3,250 g	8/9	Cesarean	Positive RT-PCR in th oropharynx swab – newborn 36 hours after delivery.	8 hours of maternal symptoms + 36 hours postpartum	Not justified: RT-PCR the in oropharynx swab collected after more than 12 hours of delivery.
#2 Dong et al. 10	China; Wuhan; Renmin Hospitalof Wuhan University	29	37weeks 6 days	3,120 g	9/10	Cesarean	Positive IgM and IgG 2 hours after delivery; negative RT-PCR in the oropharynx swab; IgM and IgG levels were reduced after 2 weeks; discharged.	25 days	Not justified: discordance among exams and negative RT-PCR in the oropharynx swab.
#3 Zamaniyan et al. 11	Iran; Sari; Imam Khomeini Hospital	22	32 weeks	2,350 g	8/9	Cesarean	Positive RT-PCR in the amniotic fluid collected during cesarean section (contamination?); negative RT-PCR in the oropharynx swab of the newborn; maternal death postpartum.	7 days	Not justified: positive RT-PCR in the amniotic fluid, but with the possibility of contamination with no other confirmation of the virus.
#4 Li et al. 5 and Ferrazzi et al. 12	China; Wuhan; Hubei Provincial Maternal and Child Health Center; 5 and Italy; Lombardy and Units of Padua and Modena 12	NR	NR	NR	NR	Vaginal	Positive RT-PCR in two newborns. In these two cases, because viral testing was not performed immediately after birth, postpartum transmission cannot be excluded.	Not informed	Not justified: the swab test was not performed shortly after birth.
#6 Alzamora et al. 13	Peru; Lima; British American Hospital	41	33 weeks	2,970 g	6/8	Cesarean	Positive RT-PCR in the nasopharyngeal swab – newborn 16 hours after delivery. Repeated in 48 hours: also positive.	5 days	Not justified: RT-PCR in the nasopharyngeal swab collected 12 hours after delivery.
#7 Vivanti et al. 14	France; Paris Saclay University Hospitals	23	35 weeks 5 days	2,540 g	4/1	Cesarean	Amniotic fluid collected during cesarean section positive on RT-PCR (Genes E and S). Nasopharyngeal and rectal swabs collected within 1 hour, 3 days and 18 days of life were positive on RT-PCR (Genes E and S). Blood and non-bronchoscopic bronchoalveolar lavage fluid collected before extubation (6 hours after delivery) positive on RT-PCR (Genes E and S).	5 days	Justified: positive RT-PCR in the amniotic fluid and in the neonate blood sample collected before 12 hours of delivery.
#8 Mehta et al. 15	United States; Liningston; Saint Barnabas Medical Center	39	27 weeks	Baby A: 925 g; Baby B: 1,050 g	Baby A: 1/3; Baby B: 5/6	Cesarean	Twin A tested positive and twin B tested negative for SARS-CoV-2 at 72 hours, and it is not clear what type of test was performed. The authors believe that twin A tested positive due to vertical transmission.	14 days	Not justified: the type of test performed is not described properly, and it was performed 12 hours after delivery.
#9 Sun et al. 16	China; Henan; Henan Provincial People's Hospital	28	37 weeks	Not informed	9/9	Cesarean	Obstetricians, anesthesiologists, neonatologists, and nurses wore full personal protective equipment, including an N95 mask, eye goggles, face shield, and a top-to-bottom tight-fitting gown, entering the operating theaters ∼ 5 minutes before the patients. Neonate tested positive for SARS-CoV-2 at day 6 postpartum.	9 days of maternal symptoms + 6 postnatal days	Not justified: RT-PCR in the oropharynx swab collected 12 hours after delivery.

Abbreviations: IgG, immunoglobulin G; IgM, immuniglobulin M; NR, not reported; RT-PCR, real-time polymerase chain reaction; SARS-CoV-2, severe acute respiratory syndrome coronavirus 2.

### Seventeen Systematic Reviews ( Box 1 )

**Box 1:** Conclusions from systematic reviews, narrative reviews and large observational studies identified in the present review

Systematic reviews
Di Mascio et al. (2020): 17 “In mothers infected with coronavirus infections, including COVID-19, > 90% of whom also had pneumonia, PTB is the most common adverse pregnancy outcome. Miscarriage, preeclampsia, cesarean, and perinatal death (7-11%) were also more common than in the general population. There have been no published cases of clinical evidence of vertical transmission.”
Della Gatta et al. (2020): 18 “The available data on COVID-19 illness in pregnant patients do not provide a clear conclusion into the clinical implications for mother and fetus. The outcome thus far described is favorable, but fetal and maternal risks should be underestimated. Although preterm delivery was mostly the consequence of elective interventions, a trend towards spontaneous prematurity is present. It is essential that future studies provide more detailed information on maternal and fetal conditions, as well as the rationale for obstetric interventions. Experience, thus far, is limited to patients that developed the disease in late gestation and were delivered shortly after the diagnosis. The fetal consequences of long-standing infections occurring in early gestation are unknown.”
Zaigham and Andersson (2020): 19 “Current evidence suggests the possibility of severe maternal morbidity requiring ICU admission and perinatal death with COVID-19 infection in pregnancy. Maternal-fetal transmission of the SARSCoV-2 virus was not detected in the majority of the reported cases, although one neonate had a positive qRT-PCR 36 hours after birth despite being isolated from the mother. Careful monitoring of pregnancies with COVID-19 and measures to prevent neonatal infection are warranted.”
Abdollahpour and Khadivzadeh (2020): 20 “No trustworthy evidence is available yet to support the possibility of vertical transmission of COVID-19 infection from the mother–baby. Mother-to-child transmission of respiratory viruses mostly happens via the birth canal and during breastfeeding or close contact among health care providers, family members. To our knowledge, no article has reconnoitered that reporting a newly vertically transmitted case.”
Banaei et al. (2020): 21 “There was some evidence about neonates COVID-19 in the included studies, but it is not clear whether the source of the infection in these neonates is from the mother or from the environment. In the majority of studies, there was no evidence of vertical transmission. In most studies, the neonates were separated from the mother after birth to reduce the chance of transmission, but there is also currently insufficient evidence regarding the mother/baby separation. If the mother is severely or critically ill, separation should be considered. The result of a review showed that in SARS vertical transmissions were not seen.
Duran et al. (2020): 22 “There is still no evidence supporting vertical transmission of COVID-19. Some newborns were positive for COVID-19 in spite of the reported use of preventive measures during and after delivery, but even in these cases there was no evidence supporting vertical transmission.”
Elshafeey et al. (2020): 23 “We extracted data regarding potential vertical transmission. In four neonates who had RT-PCR confirmed infection, samples from cord blood and amniotic fluid were negative. Based on the available data, we are uncertain of the mode of transmission, since there is no evidence that these four cases were the result of a vertical transmission.”
Gordon et al. (2020): 24 “Neonatal infection is uncommon, with only two previously reported cases likely to be of vertical transmission. The case we report is still RT-PCR-positive on day 28, and is asymptomatic.”
Huntley et al. (2020): 25 “Data from early in the pandemic is reassuring that there are low rates of maternal and neonatal mortality and vertical transmission with SARS-CoV-2.”
Juan et al. (2020): 26 “Despite the increasing number of published studies on COVID-19 in pregnancy, there are insufficient good-quality data to draw unbiased conclusions with regard to the severity of the disease or specific complications of COVID-19 in pregnant women, as well as vertical transmission, perinatal and neonatal complications.”
Kasraeian et al. (2020): 27 “Currently, no evidence of vertical transmission has been suggested at least in late pregnancy. No hazards have been detected for fetuses or neonates. Although pregnant women are at an immunosuppressive state due to the physiological changes during pregnancy, most patients suffered from mild or moderate COVID-19 pneumonia with no pregnancy loss, proposing a similar pattern of the clinical characteristics of COVID-19 pneumonia to that of other adult populations.”
Ludvigsson (2020): 28 “Newborn infants have developed symptomatic COVID-19, but evidence of vertical intrauterine transmission was scarce.”
Muhidin et al. (2020): 29 “No fetal infection through intrauterine vertical transmission was reported.”
Smith et al. (2020) 30 : “It is unclear if this is evidence of vertical transmission or if it was contracted post-delivery due to delayed RT-PCR testing 36 hours from birth. The evidence for vertical transmission appears equivocal.”
Walker et al. (2020): 31 “To date, there have been 28 cases published where the possibility for vertical transmission to have occurred have been reported. To confirm definite vertical transmission, it has been proposed that detection of the virus by PCR in either umbilical cord blood, neonatal blood collected within the first 12 hours of birth, or amniotic fluid collected prior to rupture of membranes is needed. In no cases reported to date have these criteria been met although some report negative testing. A few cases deserve special mention: one case reports a positive nasopharyngeal swab in the neonate on the day of birth. The authors do not describe any procedure or care taken to clean the infant's oropharynx / mouth/nares / face prior to procuring the swab and we speculate that the presence of the virus may be due to contamination by maternal stool. Of note, the virus was not detected on repeat swab and the infant remained well. The presence of IgG would be maternal, so again not diagnostic. The UKOSS study reports 12/24 cases of possible vertical transmission. Limited information is given for the 12 neonates but 6/12 infants tested positive for COVID-19 within 12 hours of birth. It is unclear what method of testing was used and if this was a nasopharyngeal swab without precautions to clean the infant prior to testing, may again be a result of contamination. In another case, a positive nasopharyngeal swab in the neonate on the day of birth occurred after careful separation of the baby and cleansing of the baby prior to taking the swab.”
Yang and Liu (2020) 32 : “There is currently no direct evidence to support intrauterine vertical transmission of SARS-CoV-2. Additional RT-PCR tests on amniotic fluid, placenta, and cord blood are needed to ascertain the possibility of intrauterine vertical transmission. For pregnant women infected during their first and second trimesters, further studies focusing on long-term outcomes are needed.”
Yang et al. (2020): 33 “Currently, there is no direct evidence suggesting that COVID-19 in pregnancy could lead to fetal infection via intrauterine vertical transmission. Long-term outcomes and potential intrauterine vertical transmission need further analysis.”

Abbreviations: ICU, intensive care unit; IgG, immunoglobulin G; PTB, preterm birth; qRT-PCR, real-time quantitative polymerase chain reaction; RT-PCR, real-time polymerase chain reaction; SARS, severe acute respiratory syndrome ; SARS-CoV-2, severe acute respiratory syndrome coronavirus 2; UKOSS, United Kingdom Obstetric Surveillance System.

We found a total of 17 systematic reviews:

- One systematic review including 79 pregnancies based on 19 studies from China, Saudi Arabia, South Korea, the United Arab Emirates, Jordan, Canada, Hong Kong, and the US. 17
- One systematic review including 51 pregnancies based on 6 studies from China. 18
- One systematic review including 108 pregnancies based on 18 studies from China, Sweden, South Korea, and Honduras. 19
- One systematic review not reporting the number of cases, based on 29 studies from China, Iran, France, and the US. 20
- One systematic review including 123 cases based on 16 studies from China. 21
- One systematic review including 222 cases based on 17 studies from China, Australia, Iran, and Spain. 22
- One systematic review including 385 pregnancies and 256 newborns based on 33 studies from China, Australia, Honduras, Iran, South Korea, Sweden, Turkey, Italy, The Netherlands, and the US. 23
- One systematic review including 46 cases based on 8 studies from China, Belgium, Spain, Iran, and Peru. 24
- One systematic review including 538 pregnancies based on 13 studies from China, Italy, and the US. 25
- One systematic review including 324 pregnancies based on 24 studies from China, Iran, the US, Italy, Spain, Peru, Sweden, Turkey, South Korea, Australia, Canada, and France. 26
- One systematic review including 87 pregnancies based on 9 studies from China and Iran. 27
- One systematic review including 89 pregnancies based on 9 studies from China. 28
- One systematic review including 89 pregnancies based on 9 studies from China. 29
- One systematic review including 92 pregnancies based on 9 studies from China. 30
- One systematic review including 665 pregnancies based on 49 studies from China. 31
- One systematic review including 83 neonates based on 22 studies from China, Peru, South Korea, and Spain. 32
- One systematic review including 114 pregnancies based on 18 studies from China. 33

## Discussion

Most studies published so far have registered the absence of vertical transmission, few cases of possible vertical transmission, and exceptionally, one case described as confirmed transplacental transmission of SARS-CoV-2 infection. 14 The identified cases were reported as case reports; therefore, there is a very high risk of selection bias. After defining the eighteen eligible articles, we identified only seven suspected cases of possible vertical transmission: two cases in China, one in Iran, two in Italy, one in Peru, and one in France ( Table 1 ).

The first are three different reports 7 8 9 on the same case report from China that present a methodology bias, since the collection of the newborn's oropharynx swab for the polymerase chain reaction (PCR) test was not performed at the time of delivery, which does not unequivocally guarantee the occurrence of vertical transmission. In the second Chinese case, 10 the diagnosis of possible vertical transmission was made by positive immunoglobulin M (IgM) testing two hours after delivery, but was followed by PCR swab tests that did not identify the virus in the neonate's pharynx. In the reported case from Iran, 11 the virus in the amniotic fluid was identified by PCR during the cesarean section, but there was no positivity in the nasopharynx samples, which suggests the possibility of perioperative contamination. 11 Within the two reported cases in Italy, in which the swab was not performed shortly after birth, the authors themselves suggest the possibility of postpartum transmission. 12 The case 13 reported in Peru showed positivity for the virus by PCR in the sample collected after 16 hours, and in the controls 48 hours later. In this case report, 13 the patient presented a positive PCR with a short interval between diagnosis and delivery, but the authors did not evaluate different tissues, nor performed serological tests. More recently, there was another report 15 of a case of a twin pregnancy in a medical center in the United States. Twin A tested positive 72 hours after birth; however, she did not exhibit any symptoms of infection. Twin B tested negative 72 hours after birth. The authors argue that ,because the patient was intubated at the time of delivery, that would make droplet transmission unlikely. Moreover, they claim that, because the babies were delivered via cesarean section, that would eliminate the possibility of fetal contact with maternal feces, which has been reported as a mode of transmission. Maternal contact with the neonates was avoided, they were not breastfed, and appropriate aerosol and contact precautions were taken during their handling in the neonatal intensive care unit (NICU). Due to the aforementioned reasonse, they believe that Twin A tested positive due to vertical transmission. There are crucial limitations to this theory. First, the placenta and umbilical cord blood were not tested for COVID-19, and, second, vertical transmission would have affected both twins, but Twin B, in that case, tested negative. Another article recently published with regards to possible evidence of mother-to-newborn COVID-19 infection reported a case of a neonate that tested positive for SARS-CoV-2 at ay 6 post-partum. In this specific case, obstetricians, anesthesiologists, neonatologists, and nurses wore full personal protective equipment (PPE), including an N95 mask, eye goggles, face shield, and a top-to-bottom tight-fitting gown, entering the operating theaters ∼ 5 minutes before the patients. However, the newborn was discharged home 11h after birth, and tested positive for SARS-CoV-2 on postnatal day (PND) 1. Three days later, his caregiver (his grandmother) also tested positive for SARS-CoV-2. In this case, it is not possible to establish vertical transmission as the route of contamination, once the newborn had contact with other potentially-infected people. 16 According to a recently published classification system 34 for the definition of SARS-CoV-2 infection in pregnant women, fetuses, and neonates, a neonatal congenital infection is considered confirmed if the PCR detects the virus in the amniotic fluid collected before the rupture of the membrane or in the umbilical cord blood or neonatal blood collected within the first 12 hours of birth.

The only case 14 with sufficient evidence of vertical transmission was reported in France: a pregnant woman, 23 years old, 35 weeks of gestational age, was admitted with fever and severe cough, and diagnosed with SARS-CoV-2 through real-time quantitative PCR (qRT-PCR) analysis (genes E and S) of blood and swab (vaginal and nasopharinx). She delivered through cesarean section due to acute fetal distress, and presented a positive result in the amniotic fluid on the qRT-PCR (genes E and S). In the diagnosis of the newborn, nasopharyngeal and rectal swabs were collected within 1 hour, 3 days, and 18 days of life, and were found positive on the qRT-PCR (genes E and S). Blood and non-bronchoscopic bronchoalveolar lavage fluid collected before extubation (6 hours after delivery) were also positive the on qRT-PCR (genes E and S). According to the study, 14 the samples were properly collected. Within the first few days of life, the newborn presented neurological symptoms and impairment on a magnetic resonance scan of the central nervous system similar to those described for adults in a previous study. 35 The histological examination of the placenta showed a severe inflammatory process, and the qRT-PCR was extremely positive for both SARS-CoV-2 genes, suggesting placental transmission.

Considering the interval between maternal infection and the alleged vertical transmission, the information varies considerably. So far, conventional knowledge dictates that the placenta delays transmission for maternal viral infection; however, a recently-published study demonstrated that the ACE2 and the transmembrane protease serine 2 (TMPRSS2) are expressed in human trophectoderm and placentas throughout the 3 trimesters of pregnancy. 36 Therefore, the cells of the trophectoderm and the placenta should also be considered target sites for this coronavirus infection. This might suggest that pregnancies complicated with COVID-19 are potentially at risk of intrauterine fetal or placental SARS-CoV-2 infection. 36 This information was obtained through a bioinformatic analysis, and the immunohistochemical experiments were based on a limited number of samples. Considering the impact of individual heterogeneity, further analyses are required to investigate whether the expression patterns of ACE2 and TMPRSS2 can be extended to the general population.

On the other hand, a recent study 37 evaluating the protein expression of ACE2, both in placentas and fetal organs from non-infected pregnancies throughout gestation, has observed the absence of ACE2 expression in the fetal brain and heart. This is reassuring regarding the risk of congenital malformation, but the clinical follow-up of infected pregnant women and their children is required to validate these observations. 37 With regards to vertical transmission, what we have observed so far is that the risk is very low. The mechanisms that might be involved in the maternal-fetus transmission are not clear.

Among the systematic reviews that were eligible for the present study, a total of 238 pregnancies and 174 deliveries were evaluated, and only 1 case of suspected vertical transmission, which was also mentioned in the present review (case #7– Table 1 ), was identified.

## Conclusion

The risk of vertical transmission by SARS-CoV-2 is probably very low. Despite millions of confirmed cases of COVID-19 worldwide, which probably include several thousands of pregnant women, we have identified only 1 case that has fulfilled sufficient criteria to be nominated as a confirmed vertical transmission. Well-designed observational studies evaluating large samples are still necessary to determine the risk of vertical transmission depending on the gestational age at infection. Additionally, we also need large observational studies to evaluate whether the infection by SARS-CoV-2 during pregnancy is related to an increased risk of adverse obstetrical outcomes or birth defects.
